# Supplementary material for: Visual impairment and refractive error in school children in Bhutan: The findings from the Bhutan School Sight Survey (BSSS 2019)
Source: PLoS One. 2020 Sep 14;15(9):e0239117. doi: 10.1371/journal.pone.0239117 (PMC7489552; doi:10.1371/journal.pone.0239117)
Supplement: S1 Table — (DOCX) [file pone.0239117.s001.docx]

**S1 Table.** Distribution of enumerated and examined populations by age, gender, class-level, school location, student type and parental education.

|  | **Enumerated Population** | | **Examined Population** |
| --- | --- | --- | --- |
|  | No. (%) | % Examined | No. (%) |
| Age (yrs) |  | | |
| 9-10 | 628 (12.4) | 99.2 | 623 (12.5) |
| 11-12 | 1543 (30.5) | 98.9 | 1526 (30.6) |
| 13-14 | 1504 (29.7) | 98.7 | 1484 (29.8) |
| 15-16 | 1068 (21.1) | 97.7 | 1043 (20.9) |
| 17-18 | 317 (6.26) | 97.5 | 309 (6.20) |
| Gender |  | | |
| Male | 2455 (48.5) | 98.4 | 2416 (48.5) |
| Female | 2605 (51.5) | 98.6 | 2569 (51.5) |
| Class-Level |  | | |
| IV | 764 (15.1) | 98.8 | 755 (15.2) |
| V | 816 (16.1) | 99.1 | 809 (16.2) |
| VI | 811 (16.0) | 98.6 | 800 (16.1) |
| VII | 854 (16.9) | 99.1 | 846 (17.0) |
| VIII | 877 (17.3) | 97.7 | 857 (17.2) |
| IX | 938 (18.5) | 97.9 | 918 (18.4) |
| School Location |  | | |
| Rural | 2689 (53.1) | 98.7 | 2653 (53.2) |
| Urban | 2371 (46.9) | 98.4 | 2332 (46.8) |
| Student Type |  | | |
| Day | 3614 (71.4) | 98.5 | 3559 (71.4) |
| Boarder | 1446 (28.6) | 98.6 | 1426 (28.6) |
| Parental Education |  | | |
| None | 1936 (38.3) | 99.9 | 1934 (38.8) |
| Primary | 1110 (21.9) | 99.6 | 1105 (22.2) |
| Low/Mid Secondary | 807 (16.0) | 100.0 | 807 (16.2) |
| Higher Secondary | 649 (12.8) | 99.7 | 647 (13.0) |
| Tertiary | 441 (8.72) | 99.8 | 440 (8.83) |
| Missing Information | 117 (2.31) | 44.4 | 52 (1.04) |
| All | 5060 (100.0) | 98.5 | 4985 (100.0) |
