# Supplementary material for: Visual impairment and refractive error in school children in Bhutan: The findings from the Bhutan School Sight Survey (BSSS 2019)
Source: PLoS One. 2020 Sep 14;15(9):e0239117. doi: 10.1371/journal.pone.0239117 (PMC7489552; doi:10.1371/journal.pone.0239117)
Supplement: S2 Table — (DOCX) [file pone.0239117.s002.docx]

**S2 Table.** Prevalence of astigmatism by age and gender.

|  | Number of Children | **Astigmatism Severity*** | | | |
| --- | --- | --- | --- | --- | --- |
|  |  | **Mild^†^** | **Moderate^‡^** | **High^‖^** | **Any** |
| Age (yrs) |  |  | | | |
| 8-10 | 623 | 33 (5.30) | 6 (0.96) | 16 (2.57) | 55 (8.83) |
| 11-12 | 1526 | 96 (6.29) | 22 (1.44) | 39 (2.56) | 157 (10.3) |
| 13-14 | 1484 | 85 (5.73) | 18 (1.21) | 38 (2.56) | 141 (9.50) |
| 15-16 | 1043 | 74 (7.09) | 8 (0.77) | 20 (1.92) | 102 (9.78) |
| 17-18 | 309 | 20 (6.47) | 5 (1.62) | 6 (1.94) | 31 (10.0) |
| Gender |  |  | | | |
| Male | 2416 | 139 (5.75) | 29 (1.20) | 51 (2.11) | 219 (9.06) |
| Female | 2569 | 169 (6.58) | 30 (1.17) | 68 (2.65) | 267 (10.4) |
| All | 4985 | 308 (6.18) | 59 (1.18) | 119 (2.39) | 486 (9.75) |

Data are presented as number (%) of children.

* Severity is categorized using the cylinder value in the eye with the greatest astigmatism.

^†^ ≥0.75 to <1.50 diopters.

^‡^ ≥1.50 to <2.00 diopters.

^‖^ ≥2.00 diopters.
