# Supplementary material for: Visual impairment and refractive error in school children in Bhutan: The findings from the Bhutan School Sight Survey (BSSS 2019)
Source: PLoS One. 2020 Sep 14;15(9):e0239117. doi: 10.1371/journal.pone.0239117 (PMC7489552; doi:10.1371/journal.pone.0239117)
Supplement: Attachment — Submitted filename: PONE-D-20-11483.pdf [file pone.0239117.s004.pdf]

**Reviewer comments to Authors**

Overall the paper needs improvements throughout all sections. However, the results and discussion sections are the ones that need major improvement, as the message it's no clear. The sections need to be reformulated to emphasize the visual impairment based on best corrected VA and not under corrected VA. The authors can also highlight the importance of present VA to show that many children are not well corrected or need glasses. Looking at visual impairment using best corrected VA we can see that the number of visual impaired children is very low. The number of uncorrected refractive cases is high, and this is a different concept that needs to be explained. Another important concept that needs explanation is the use of "prevalence of myopia" determined with cycloplegia. This does not look the most adequate for this study and might be misinterpreted, as only a subset of children with VA of 6/12 or below were properly refracted with cycloplegia and not all children.

**Abstract**

Line 51. The definition for astigmatism is given, but not for myopia and hyperopia.

**Introduction**

Line 71 to 77. This section does not look relevant to this paper. The introduction needs to highlight the publications on the topic and relevance of this study.

Line 82 to 84. This sentence looks misplaced. Maybe this sentence would fit better in the discussion section.

Line 84 to 90. This section belongs to the methods and not the introduction.

Methodology

The Helsinki declaration should be added as this is research conducted with human subjects.

Line 113 to 114, Line 118 to 125 and Line 128 should be deleted.

Line 131 – Children were 10 and 16 years of age, so it is unclear why was the tumbling-E optotypes used?

Line 139 - What was the rationale for the distance used in the cover test (0.5 and 4.0 meters)? Those are not the conventional distances and a citation would be appreciated.

Line 152 to 153 - This is not the scope of a scientific paper. One sentence to say they were referred, and glasses were given, is enough.

Line 157 to 161 - Combine this section with the one on the informed consent.

Line 169 – The focus of the paper needs to change. Why was visual impairment calculated from the uncorrected VA? Shouldn't this be only for the best correct VA?

Lines 176 to 178 - Include astigmatism definition as this is only referred in the abstract and results section.

Lines 178 and 181 – This is a huge limitation of this paper and it is not properly addressed in the discussion section.

Results

Line 196. This is a repetition of the methods section. Do not repeat information.

Line 201. This sentence should be in the methods section, not in the results.

Line 208 and 209. Please, add information on the standard deviation.

Discussion

There is not enough discussion. How this prevalence compares with other Asian regions? What about future studies that need to be conducted?

Line 344 to 346. Is there any study that proves this? Please, include citation.
