# Supplementary material for: Visual impairment and refractive error in school children in Bhutan: The findings from the Bhutan School Sight Survey (BSSS 2019)
Source: PLoS One. 2020 Sep 14;15(9):e0239117. doi: 10.1371/journal.pone.0239117 (PMC7489552; doi:10.1371/journal.pone.0239117)
Supplement: Attachment — Submitted filename: Response to Reviewers.docx [file pone.0239117.s005.docx]

**Response to Reviewers**

**Title: Visual impairment and refractive error in school children in Bhutan: The findings from the Bhutan School Sight Survey (BSSS 2019)**

**Authors:** Indra Prasad Sharma, Nor Tshering Lepcha, Tshering Lhamo, Leon B Ellwein, Gopal Prasad Pokharel, Taraprasad Das, Yuddha Dhoj Sapkota, Tandin Dorji, Sonam Peldon

**Ref.: PONE-D-20-11483**

**Reviewer# 1**

Overall the paper needs improvements throughout all sections. However, the results and discussion sections are the ones that need major improvement, as the message it’s no clear. The sections need to be reformulated to emphasize the visual impairment based on best corrected VA and not under corrected VA. The authors can also highlight the importance of present VA to show that many children are not well corrected or need glasses. Looking at visual impairment using best corrected VA we can see that the number of visual impaired children is very low.

The number of uncorrected refractive cases is high, and this is a different concept that needs to be explained. Another important concept that needs explanation is the use of “prevalence of myopia” determined with cycloplegia. This does not look the most adequate for this study and might be misinterpreted, as only a subset of children with VA of 6/12 or below were properly refracted with cycloplegia and not all children.

**Response:** Thank you for your review and comments. We have responded to all the comments below.

| **Location** | **Reviewer’s comments** | **Reply from author(s)** |
| --- | --- | --- |
| Abstract |  |  |
| Line 51 | Definition of astigmatism given, not myopia and hyperopia | Definition of myopia nd hyperopia is now added; lines 47 and 49. |
| Introduction |  |  |
| Lines 71-77 | This section does not look relevant to this paper. The introduction needs to highlight the publications on the topic and relevance of this study. | Lines 71-77 are deleted.  The publications on the subject in different parts of the world and relevance of the current study was already mentioned briefly earlier. Now its elaborated in the ‘Introduction’ |
| Lines 82-84 | This sentence looks misplaced. Maybe this sentence would fit better in the Discussion section | These are shifted to ‘Discussion’ section. Lines 321-324. |
| Lines 84-90 | This section belongs to Methods and not the Intoduction. | Part of the sentence was retained and part (lines 88-90) were shifted to Methods section. |
| Methodology | The Helsinki Declaration should be addded as this is research conducted with humam subjects. | Thanks. It is added.  Lines 133-134 |
| Lines 113-114  Lines 118-125 | Should be deleted | These sentences describe an important part of methodology. We have abridged the sentences and request to retain the new ones. Lines 136-137 and Lines 137-141 |
| Line 131 | Children were 10 and 16 years of age; so it is unclear why Tumbling E optotypes were used? | The reasons are now explained in lines 147-148. “Tumbling optotypes were used so that the children do not memorize the letters.” Tumbling optotypes were also used in the other RESC surveys. |
| Line 139 | What was the rationale for the distance used in cover test (0.5 and 4.0 m)? Those are not the conventional distances and a citation would be appreciated. | Thanks. This was a mistake. Cover test was measured at 0.5m (for near instead of 33-40 cms) and 6.0m (for distance) This is now corrected; Line 154-155. |
| Lines 152-153 | This is not the scope of the scientific paper. One sentence to say they were referred and glasses were given is enough. | This now corrected. Lines 166-167. |
| Lines 157-161 | Combine this section with one with the one on the informed consent. | Done. Lines 129-133 |
| Line 169 | The focus of the paper needs to change. Why was visual impairment calculated from the uncorrected VA? Shouldn’t this be only for best corrected visual acuity? | UCVA was the entire basis upon which refractive error was measured; and thus, central to our data analysis. Further, by comparing PVA with UCVA and BCVA, is was possible to determine if the subject was wearing the right spectacles. Hence we will like to retain this sentence. |
| Lines 176-178 | Include astigmatism definition as this is only referred in the abstract and results section. | Thanks. This is now added. Lines 188 |
| Lines 178-181 | This is a huge limitation of this paper and is not properly addressed in the Discussion section | We present data on the prevalence of visual impairment associated with hyperopia and myopia. Because we did not obtain cycloplegic refraction data in children with normal/near-normal vision in both eyes, the prevalence of ametropia is based on children with visual impairment. This has been restated for clarification in the Methods section.  It is now noted in the Discussion section that “to the extent that some children with normal or near normal vision may have been ametropic, our data are likely to underestimate the actual prevalence; representing a limitation of the RESC protocol.”  Lines 345-347 |
| Results |  |  |
| Line 196 | This is repetition of the Methds section. Do not repeat information. | Thanks. This sentence is now deleted. |
| Line 201 | This sentence should be in Methods section, not in Results | This sentence is now shifted to Methods section; Line 141. |
| Lines 208-209 | Please, add information on the standard deviation. | Standard deviations have now been added. Now in Lines 222-223 |
| Discussion | There is not enough Discussion. How is this prevalence compares with other Asian regions? What about future studies that need to be conducted? | The ‘Discussion’ is rewritten. Comparison is done; Lines 328-337. Future studies are suggested; Lines 375-376 |
| Lines 344-346 | Is there any study that proves this? Please, include citation. | The sentence is reconstructed and reference cited; Lines 356-357 Reference #26. |

**Reviewer #2**

Visual impairment (VI) rate has yet been well reported in Bhutan. In this regard, this study provides good value-add.

Response: Thank you for your review and comments. We have

| **Question #.** | **Reviewer’s comments** | **Reply from author(s)** |
| --- | --- | --- |
| 1. | Why did the authors focus on uncorrected VI rather than presenting VI? I would think presenting VI is more relevant/ important. | In our opinion measurement of both uncorrected and presenting (corrected with the current spectacles) is necessary to identify if the subject was wearing the right spectacles. |
| 2. | To be more consistent with US definition of VI: I would suggest the authors to define VI as <6/12 rather than ≤6/12. | To the extent that some children with normal/near normal vision may have been ametropic, ≤6/12 would be a better cut-off. Defined as per the RESC protocol. |
| 3. | The hyperopia definition of +2.00 D or more seems rather stringent, and may inadvertently underestimate the true prevalence of hyperopia. Why not define as +1.00D or even +0.50 dioptre? Any reason for this? Given that Bhutan is at higher altitude, is there a higher prevalence of hyperopia compared to other countries? | We followed the RESC protocol definition for hypermetropia. In our opinion, hypermetropia of less than +2.00D is less likely to cause visual impairment. Hyperopia prevalence was similar to those reported in other RESC studies. |
| 4. | I believe this is one of the first reports of such from Bhutan – it would be nice if the authors can discuss and compare these findings with other previous population-based studies from other countries/ regions. And from there – perhaps set the scope for future public health, interventional directions on visual impairment prevention for school children in Bhutan. | Thank you. We have made changes the discussion section as suggested. Table 5 is added to show the results of previous RESC studies. |
| 5. | It would be good if the authors can also present age-standardised prevalence – standardized to national population census (for that age group) if possible. | National census data are available for age groups that do not correspond with the age-specific study data categories. Further, because the study sample was drawn nationally across all Bhutan, the prevalence estimates (with 95% CI) already pertain to the entire national population |
| 6. | In addition to the current Table 3 – perhaps it is also meaningful to evaluate factors associated with presenting visual impairment. | Table 3 addressed hyperopia and myopia in all children with visual impairment. Leaving out the 73 children who corrected to normal/near-normal with current spectacles would have resulted in an incomplete representation of refractive error in children with VI. (Further, because so few corrected with spectacles the findings would have been essentially the same.) |
