# Supplementary material for: Visual impairment and refractive error in school children in Bhutan: The findings from the Bhutan School Sight Survey (BSSS 2019)
Source: PLoS One. 2020 Sep 14;15(9):e0239117. doi: 10.1371/journal.pone.0239117 (PMC7489552; doi:10.1371/journal.pone.0239117)
Supplement: Attachment — Submitted filename: Response to Reviewers.docx [file pone.0239117.s006.docx]

**Response to Reviewers**

**Title: Visual impairment and refractive error in school children in Bhutan: The findings from the Bhutan School Sight Survey (BSSS 2019)**

**Authors:** Indra Prasad Sharma, Nor Tshering Lepcha, Tshering Lhamo, Leon B Ellwein, Gopal Prasad Pokharel, Taraprasad Das, Yuddha Dhoj Sapkota, Tandin Dorji, Sonam Peldon

**Ref.: PONE-D-20-11483 R1**

**Reviewer #1:**

***Reviewer’s comment:*** The paper is much improved with the revisions done by the authors. However, I still feel that visual impairment results should rely on best corrected VA and not under corrected VA. The prevalence of visual impairment using best corrected VA is very low (only 0.34%) and this finding is ignored in the discussion section. The paper would benefit from having two sections in the results, 1) highlight the importance of present VA (compared to uncorrected VA) to show that many children are not well corrected or need glasses; The number of uncorrected refractive cases is high. 2) Visual impairment based on best corrected VA.

***Reply from Authors:*** Thank you for your comments. Visual impairment results for uncorrected, presenting, and best corrected VA are presented in Table 1 in the Results Section. A new paragraph is added (Lines 335-342) as suggested by the reviewer.

**Reviewer #2**

***Reviewer’s comment:*** All previous comments have been well addressed by the authors during this current revision.

***Reply from Authors*:** Thank you.
